# Protein Deposition on Sport Mouthguards and the Effectiveness of Two Different Cleaning Protocols

**DOI:** 10.3390/jcm13113023

**Published:** 2024-05-21

**Authors:** Kirsten van Vliet, Annina van Splunter, Jan de Lange, Frank Lobbezoo, Henk Brand

**Affiliations:** 1 Academic Centre for Dentistry (ACTA)—Academic Medical Center Amsterdam (UMC), Department of Oral and Maxillofacial Surgery, 1081 HV Amsterdam, The Netherlands; 2 Academic Centre for Dentistry (ACTA), Department of Oral Biochemistry, 1081 LA Amsterdam, The Netherlands; 3 Academic Centre for Dentistry (ACTA), Department of Orofacial Pain and Dysfunction, 1081 LA Amsterdam, The Netherlands

**Keywords:** mouthguard, adhesion, salivary proteins, cleaning strategy

## Abstract

**Objective**: To determine which salivary proteins adhere onto sport mouthguards, and to evaluate the effectiveness of different cleaning strategies in removing deposited protein. **Methods**: Fifteen healthy volunteers used a mouthguard for 1 h. The deposited salivary proteins were analyzed using gel electrophoresis and Western blotting techniques and compared with the protein composition of unstimulated saliva. In addition, the effectiveness of two different cleaning strategies to remove proteins from the mouthguards were compared: rinsing the mouthguards after use with cold tap water and cleaning the mouthguard with a soluble effervescent tablet. **Results**: Gel electrophoresis showed deposition of proteins of 50–60 kDa and 14 kDa on the mouthguards used in the mouth for 1 h. Western blotting identified these bands as amylase and lysozyme, respectively. Rinsing the mouthguard with cold tap water after use removed 91% of the total amount of deposited proteins, while cleaning with an effervescent tablet removed 99%. **Conclusions**: During the use of mouthguards, salivary proteins are deposited on their surface. Because salivary proteins can potentially affect bacterial adhesion to mouthguards, proper cleaning after use is recommended. Cleaning the mouthguard with cold tap water or using an effervescent tablet both seem to be effective strategies to remove proteins deposited on sport mouthguards.

## 1. Introduction

Mouthguards are widely used in contact sports to protect teeth from injury [1,2,3,4,5,6,7,8,9,10,11,12]. Although mouthguards are considered to be useful for this purpose, they also appear to be associated with negative health outcomes in athletes. Considerable quantities of pathogenic and opportunistic bacteria, yeast, and fungi were found on sports mouthguards used in ice hockey and football [13,14,15]. D’Ercole et al. investigated the environmental changes in the oral cavity induced by sports mouthguards by determining clinical, salivary, and bacteriological markers, before, during, and after sports practice. Their results showed that use of mouthguards increased full-mouth plaque scores and bleeding scores, and reduced buffering capacity and salivary pH, which negatively affects the protective effect of saliva [16]. Subsequent research by the same authors suggested that chlorhexidine could be effective in reducing mouthguard contamination [17]. Another study showed that sanitizing with an antimicrobial solution (NitrAdine) significantly reduced the number of microorganisms on mouthguards [18].

In contrast to microbial removal approaches, alternative strategies focused on preventing microbial colonization by pre-coating mouthguards. The growth of the pathogenic oral bacteria *Streptococcus mutans* and *Porpyromonas gingivalis* was reduced on a mouthguard with a surface of pre-reacted glass ionomer (S-PRG). However, increasing the S-PRG filler content in the mouthguards led to increased hardness, and decreased resilience, which negatively affected the protective properties of the mouthguard [19]. A similar study examined pre-impregnation of mouthguards with casein phosphopeptide (GC Tooth Mousse). The authors concluded that this positively affected the flow rate, pH, and buffering capacity of saliva [20]. These studies indicate that both cleaning the mouthguard after use and modifying the mouthguard’s surface reduce the presence of micro-organisms. Nevertheless, the factors involved in the attachment and growth of microorganisms on mouthguards remain unexplained.

The constant release of saliva by the salivary glands results in the surface of teeth and dental materials such as implants being covered with saliva components. Binding to these host-surface-associated salivary proteins will allow bacteria to attach to the surface, thus favoring their colonization and survival [21,22,23]. Likewise, we assume that salivary proteins will adhere to the surface of the mouthguard during use. Therefore, the aim of this study was to identify salivary proteins that adhere to mouthguards, determine the biological activity of clinically relevant proteins, and to explore how mouthguards can be effectively cleaned of adhering salivary proteins.

## 2. Materials and Methods

### 2.1. Participants and Sample Collection

Fifteen healthy volunteers were recruited among the staff of the dental faculty. Five participants were male and ten were female, with a mean age of 38 years (range 23 to 63). A priori sample size calculation was performed using G*Power software, version 3.1.9.4 (Heinrich-Heine-Universität Düsseldorf, Düsseldorf, Germany), assuming a 70% decrease in protein levels after cleaning. Based on an effect size of 0.75, an α of 0.05, and a power of 80%, 15 participants were needed for this comparison.

Initially, unstimulated whole saliva was collected for 5 min based on normal salivary flow rates [24]. Then, 1 mL of the unstimulated saliva was analyzed as a control. Subsequently, the participants had to wear a mouthguard for 1 h while they were doing their regular work without removing the mouthguard. Consequently, drinking and eating was not allowed. The mouthguard was constructed from transparent thermoplastic Ethylene Vinyl Acetate (EVA) (Decathlon, Amsterdam, The Netherlands B.V.), adhering to manufacturer’s instructions, while wearing gloves to prevent contamination. After being removed from the mouth, the mouthguards were stored at 4 °C for 24 h to dry. The mouthguards were dried at this temperature to limit the risk of denaturation of the deposited proteins, associated with higher temperatures.

Next, the dried mouthguards were uniformly segmented into three sections using a sterile scalpel, and from each volunteer, the three segments were randomly assigned to one of the three following conditions: (1) no cleaning, (2) rinsing with cold tap water for 1 min, and (3) treatment for 3 min in water with a soluble effervescent tablet suitable for cleaning dentures (Steradent Triple Action, Reckitt Benckiser, Hoofddorp, The Netherlands).

As negative control, and to exclude the possible contamination of the mouthguards with proteins during the manufacturing process, unused mouthguards were analyzed as well.

### 2.2. Protein Elution and Gel Electrophoresis

Each mouthguard segment was transferred to a 50 mL tube (Greiner Bio-One^TM^ CELLSTAR^TM^, Landsmeer, The Netherlands) and 10 mL acetonitrile 70% (ACN) (Biosolve, Valkeswaard, The Netherlands) was added. The tubes were placed onto a rolling plate. After constant rolling for 1 h, the mouthguard segments were removed and the tubes were placed overnight in a rotational vacuum concentrator (Christ RVC 2-25CD plus, Osterode am Harz, Germany) to evaporate the ACN. The remaining protein pellet was dissolved in sample buffer (NuPAGE^TM^, Thermo Fisher Scientific, Waltham, MA, USA) according to the standard protocols of the manufacturer. Sodium dodecyl sulfate polyacrylamide gel electrophoresis (SDS-PAGE) was performed on 4–12% Bis-Tris plus gels (NuPAGE^TM^, Thermo Fisher Scientific, Waltham, MA, USA) and executed according to manufacturer protocols with MES buffer (Thermo Fisher Scientific, Waltham, MA, USA). As a molecular weight marker, Novex pre-stained protein standard was used (Thermo Fisher Scientific, Waltham, MA, USA). After this, the gel was rinsed with distillated water, and stained with SimpleBlue SafeStain (Thermo Fisher Scientific, Waltham, MA, USA) to visualize the presenting protein bands.

### 2.3. Western Blotting

The stained 4–12% Bis-Tris plus gels containing separated proteins were first de-stained in 20% Methanol solution. After decolorization, the gels were rinsed in Demi water and transferred to nitrocellulose membranes by semi-dry blotting (Iblot Invitrogen, Thermo Fisher Scientific) according to the manufacturer’s protocol. Mouse monoclonal antibody and human amylase at a 1:1000 dilution (sc-166349, Santa Cruz biotechnology, Dallas, TX, USA) and rabbit polyclonal antibody to human lysozyme at a 1:500 dilution (A099, Dako, Glostrup, Denmark) were used as primary antibodies, while goat anti-rabbit alkaline phosphatase (Thermo Fisher Scientific, Waltham, MA, USA) and rabbit anti-mice alkaline phosphatase (Dako, Glostrup, Denmark) conjugates at 1:1000 dilution were used as secondary antibodies. For visual detection of these antibodies, the membranes were stained with SigmaFast BCIP/NBT (Sigma Aldrich, Milwaukee, WI, USA).

### 2.4. Gel Analysis and Quantification

Graphpad Prism v9.1 (Graphpad Software Inc., Boston, MA, USA) was utilized to semi-quantify the imaged SDS-PAGE gels. A lane profile plot was generated from each individual sample, with peaks representing the present proteins. The area under each peak was used as a measure of protein quantity and expressed as Pixel Intensity (PI). The total PI of the pre-stained protein marker was used to normalize results from different SDS-PAGE gels. The person who performed these measurements was not aware of the experimental conditions of the individual samples to avoid to potential bias during this semi-quantification.

### 2.5. Amylase Biological Activity

To verify the enzymatic activity of amylase after elution from a used mouthguard, we performed an additional experiment with 4 of the volunteers. The procedure for processing mouthguards was similar to that described above. However, instead of ACN, 1 mL distilled water was used to elute the proteins. Amylase activity was measured by analyzing colorimetric-based enzymatic activity. Then, 10 μL of eluted protein sample and 90 μL of amylase substrate, 2-chloro-4-nitrophenyl-α-d-maltotrioside (Sigma-Aldrich, St Louis, MO, USA), were added to a well of a 96-well ELISA microplate (655101, Greiner Bio-One B.V, Alphen aan den Rijn, The Netherlands). The substrate was cleaved by amylase into 2-chloro-4-nitrophenol and the absorbance was measured with a Multiskan™ FC Microplate Photometer (Thermo Fisher Scientific, Waltham, MA, USA) at 405 nm for 15 min. All measurements were performed in duplicate. Amylase concentrations are expressed as arbitrary units per milliliter (AU/mL).

### 2.6. Statistical Analysis

Statistical analysis was performed using Graphpad Prism v9.1 (Graphpad Software Inc., Boston, MA, USA). The differences in pixel intensity of the lanes corresponding to total protein, amylase, and lysozyme and the amylase enzymatic activity of the three different experimental conditions were statistically analyzed using the Wilcoxon test and sub-analyzed using the Friedman test. *p* values < 0.05 were considered statistically significant.

## 3. Results

### 3.1. SDS-PAGE and Western Blotting

**Figure 1:**
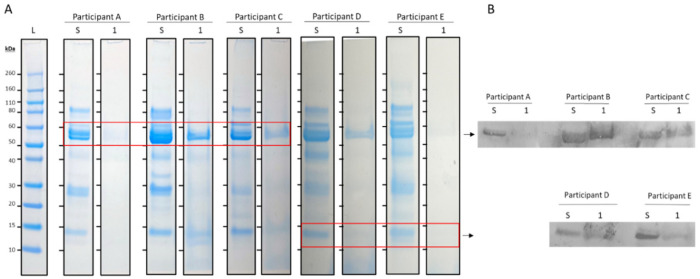
(**A**) SDS-PAGE results of unstimulated whole saliva (S) and used mouthguards without cleaning (1) from 5 different participants (A t/m E). Lane 1 (L) contains the molecular weight markers. (**B**) Western blot with antibodies against amylase (53 kDa) and lysozyme (14 kDa).

All unstimulated saliva samples showed a typical protein profile characterized by multiple protein bands on the SDS-PAGE gels. Some of these salivary proteins were also identified on the mouthguards used by the participants without cleaning (condition 1), particularly a protein of 50–60 kDa, and one with a lower molecular weight of 14 kDa (Figure 1A). Immunodetection by Western blot with specific antibodies identified the protein of 50–60 kDa as amylase and the protein of 14 kDa as lysozyme (Figure 1B).

**Figure 2:**
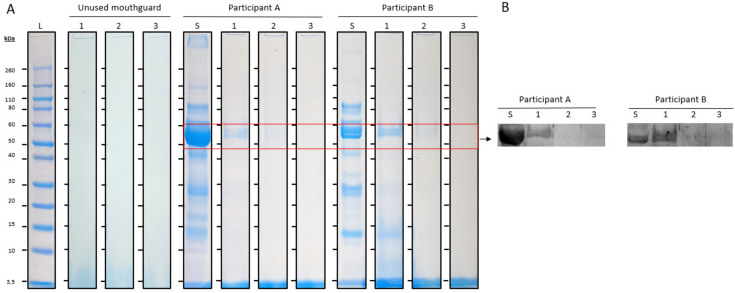
(**A**) SDS-PAGE of unused mouthguards and unstimulated saliva (S), used mouthguards without cleaning (1), mouthguards rinsed with cold tap water after use (2), and mouthguards cleaned with an effervescent tablet after use (3) from two different participants (**A**,**B**). The left lane (L) contains the molecular weight markers. (**B**) Western blot with antibody against amylase (53 kDa).

As a negative control, unused mouthguards were analyzed in comparison to used mouthguards. No proteins were identified on the unused mouthguards on the SDS-PAGE gels (Figure 2A).

The protein bands of 50–60 kDa and 14 kDa identified on the used mouthguards without cleaning (condition 1) were compared with those found on the used mouthguards rinsed with cold tap water (condition 2) and the mouthguards cleaned with an effervescent tablet (condition 3). Visually, the protein band of 50–60 kDa was much less intense in condition 2 compared to condition 1, and in condition 3 no protein bands could be observed at this molecular weight (Figure 2A). Immunodetection by Western blot confirmed the protein band of 50–60 kDa to be amylase (Figure 2B). The protein band at 14 kDa was visually barely detectable in condition 2 compared to condition 1. Condition 3 showed no protein bands at this molecular weight.

### 3.2. Gel Analysis and Quantification

**Figure 3:**
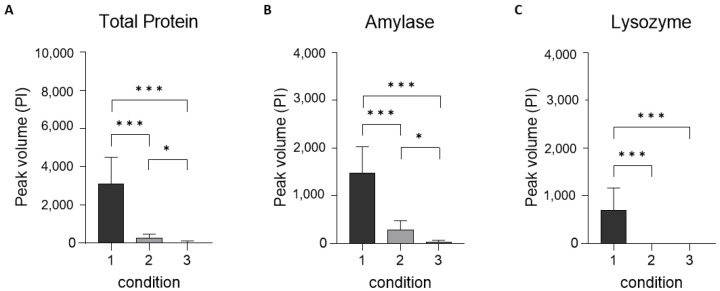
Peak Pixel Intensity of protein eluted from mouthguards without cleaning (1), mouthguards cleaned by rinsing with cold water (2), and mouthguards cleaned using an effervescent tablet (3), showing the peak volume of all proteins present (**A**), of amylase (**B**), and of lysozyme (**C**). Data are mean ± SD (*n* = 15). Level of significance: * ≤0.05, *** ≤0.001.

The SDS-PAGE gels were analyzed with Graphpad Prism v9.1 software to convert the protein bands of each condition into peak Pixel Intensities (PI). The summed PI of all adsorbed protein bands showed, on average, a 91% decrease after rinsing with cold tap water (condition 2) and a 99% decrease after cleaning with an effervescent tablet (condition 3); both indicating a significant reduction (*p* < 0.001). The effervescent tablet also significantly reduced the total number of protein bands compared to the tap water (*p* < 0.01) (Figure 3A). Rinsing with water decreased the amount of adsorbed amylase by 81% on average and cleaning with an effervescent tablet by 98%; both indicating a significant reduction (*p* < 0.001). The effervescent tablet also significantly reduced the number of amylase bands compared to the tap water (*p* <0.01) (Figure 3B). Lysozyme was no longer detectable after rinsing with tap water and after cleaning with an effervescent tablet; both indicating a significant reduction (*p* < 0.001) (Figure 3C).

**Figure 4:**
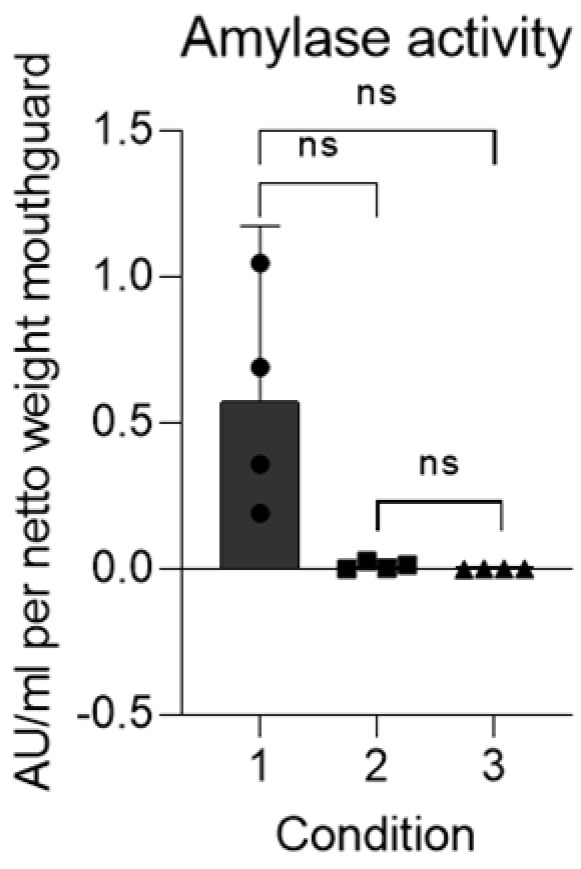
Amylase activity (in AU/mL) retrieved from mouthguards without cleaning (1), mouthguards rinsed with cold water (2), and mouthguards cleaned using an effervescent tablet (3). ns = not significant.

The amylase deposited on the mouthguards showed biological activity, which was reduced by 95% on average after rinsing with water (Figure 4). After cleaning with an effervescent tablet, no amylase activity could be detected.

## 4. Discussion

The results of this study show that salivary proteins are deposited on mouthguards while athletes wear them. The deposited salivary proteins include lysozyme and amylase.

Lysozyme is a protein that plays an important role in innate immune defense against microbial infections. It breaks down the peptidoglycan cell wall of bacteria, by hydrolyzing the glycosidic bond between N-acetylmuramic acid and N-acetylglucosamine, and thereby weakens the bacterial cell walls and ultimately leads to bacterial lysis [25,26]. For a long time, it has been assumed that lysozyme could only exert its antimicrobial activity by functioning as a hydrolytic enzyme. However, it has been shown that heat-denatured lysozyme has a sustained bactericidal activity against both Gram-positive and Gram-negative bacteria [27].

Members of the oral flora have been found to be relatively insensitive to lysis by lysozyme [28]. Given its antimicrobial role, the adsorption of lysozyme onto a mouthguard could potentially have a positive effect in maintaining oral health. However, lysozyme may bind to aggregate the pathogen *Staphylococcus aureus*, other streptococci, and putative Gram-negative periodontopathic bacteria such as *Captocytophaga gingivalis* [21,29]. Consequently, the use of unclean mouthguards could offer the pathogen a location for dissemination to other body sites. Bacteria and their byproducts in the oral cavity may lead to a wide range of systemic problems, like cardiovascular disease, kidney disease, diabetes, pulmonary disease, and different types of cancer [30].

Amylase, which was also identified on mouthguards and was even enzymatically active, plays a crucial role in the oral cavity through the digestion of carbohydrates. It cleaves large starch molecules into dextrin and subsequently into smaller maltooligosaccharides containing α-D-(1,6) linkages, the trisaccharide maltotriose, and the disaccharide maltose [31]. These sugar molecules can act as a food source for acid-producing bacteria, which can damage tooth enamel, contribute to the formation of dental plaque, and ultimately lead to dental caries [32]. The role of amylase in the oral cavity has often been considered to be of little significance due to the short duration of food in the oral cavity, but it has been shown that considerable starch hydrolysis occurs within seconds in the oral cavity [33]. Therefore, the presence of biologically active amylase on mouthguards means that a significant production of sugar molecules can occur if starch-containing foods are consumed during prolonged exercise. In addition, salivary amylase is able to bind to *Streptococcus gordonii,* a streptococcus species frequently used in vitro studies as a model organism for *S mutans* [34]. Therefore, it seems plausible that the adsorption of amylase onto sport mouthguards may provide a temporary location for *S. mutans,* from which it can colonize oral tissues.

A cross-sectional observational study in a population of 268 systemically healthy young students showed considerable variation in salivary lysozyme as well as salivary amylase concentrations [35]. In addition, in the current study the mouthguards were not worn during actual sports practice, but during resting conditions. Many studies have described the effects of physical exercise on salivary composition [36]. For instance, acute running was found to increase lysozyme secretion in saliva [37,38], even during exercise at moderate intensity [39]. Amylase also increased during exercise [40,41], although for this saliva ingredient the increase does not seem to be related to the degree of physical exercise [39]. Therefore, in follow-up studies, mouthguards should be worn during actual sports practice.

Considering this possible facilitating role of salivary proteins in bacterial binding to sport mouthguards, and the considerable quantities of micro-organisms found on sports mouthguards [13,14,15], the removal of salivary proteins from sport mouthguards after use seems to be of paramount importance. The two cleaning strategies investigated in this study revealed promising outcomes. Rinsing the mouthguards after use with cold tap water resulted in a significant reduction in all protein bands on the SDS-PAGE gels, indicating considerable protein removal. Cleaning the sport mouthguards with an effervescent tablet resulted in an even greater reduction in the amount of protein on the sport mouthguard. But, before cleaning mouthguards after use with these tablets can be recommended to athletes, the tablet’s possible effects on the surface should also be investigated. If treatment of the mouthguard with the tablets causes the surface to become rougher, this may not only reduce the athlete’s comfort during use, but a higher surface roughness may also increase biofilm formation [42].

## 5. Conclusions

Salivary proteins, including lysozyme and amylase, adhere to mouthguards. Cleaning the mouthguard with cold water or using an effervescent tablet are both effective strategies for removing proteins from mouthguard surfaces, which might be beneficial for the athlete’s health.

## Data Availability

The original contributions presented in the study are included in the article, further inquiries can be directed to the corresponding author.
